# Does the ICU experience predict psychological symptoms in relatives of patients with severe sepsis and end-of-life decisions?

**DOI:** 10.1186/cc12959

**Published:** 2013-11-05

**Authors:** Christiane Hartog, Daniel Schwarzkopf, Helga Skupin, Björn Kabisch, Ruediger Pfeifer, Albrecht Guenther, Konrad Reinhart

**Affiliations:** 1 Department of Anesthesiology and Intensive Care Medicine, Jena University Hospital, Jena, Germany; 2 Center for Sepsis Control & Care, Jena University Hospital, Jena, Germany; 3 Department of Internal Medicine I, Jena University Hospital, Jena, Germany; 4 Department of Neurology, Jena University Hospital, Jena, Germany

## Background

Severe sepsis is the main cause of death in the ICU. Relatives are at risk for post-traumatic stress disorder (PTSD) or anxiety and depression [1]. The objective is to assess whether the ICU experience may predict these psychological symptoms of relatives at 90 days after the patient's death or discharge.

## Materials and methods

Prospective observational study in four ICUs of one university hospital, including all patients with severe sepsis and end-of-life-decisions. At 90 days, the main relative was interviewed with the Impact of Event Scale (to measure PTSD), the Hospital Anxiety and Depression Scale and self-developed items on satisfaction with the ICU experience, including medical care and communication in general as well as specifically in the end-of-life context, and decision-making. Three multiple linear regression models were calculated to predict anxiety, depression and post-traumatic stress each.

## Results

Eighty-four relatives were included. They were mostly female (74%), spouse (42%) or child (42%), median age was 57 years. Seventy-seven percent acted as proxies. After 90 days, 51% relatives were at risk for PTSD, 48% for anxiety and 33% for depression. Overall satisfaction with the ICU experience was high. Relatives' satisfaction with medical care and communication in general predicted lower anxiety (*P *= 0.025).

## Conclusions

Relatives of patients with sepsis have a high psychological burden. Improving communication between ICU staff and relatives may reduce their symptoms of anxiety.
